# Digital RNA Sequencing of Human Epidermal Keratinocytes Carrying Human Papillomavirus Type 16 E7

**DOI:** 10.3389/fgene.2020.00819

**Published:** 2020-08-05

**Authors:** Chunting Hua, Jiang Zhu, Boya Zhang, Siyuan Sun, Yinjing Song, Stijn van der Veen, Hao Cheng

**Affiliations:** ^1^Department of Dermatology, Sir Run Run Shaw Hospital, School of Medicine, Zhejiang University, Hangzhou, China; ^2^Department of Microbiology and Parasitology, Collaborative Innovation Center for Diagnosis and Treatment of Infectious Diseases, School of Medicine, Zhejiang University, Hangzhou, China

**Keywords:** human epidermal keratinocytes, human papillomavirus type 16, E7 gene, AKAP12, DUSP5, MAPK signaling pathway

## Abstract

High-risk human papillomavirus (HPV) infections are the predominant cause of cervical cancer and its early gene E7 plays an important role in cellular proliferation and cell-cycle progression. While tremendous progress has been made in exploring the molecular mechanisms in late tumorigenesis, many pathways showing how HPV deregulates host gene expression in early inapparent infections and early tumorigenesis still remain undefined. Digital RNA sequencing was performed and a total of 195 differentially expressed genes were identified between the HPV16 E7-transfected NHEKs and control cells (*p* < 0.05, fold-change > 2). GO enrichment showed that HPV16 E7 primarily affected processes involved in anti-viral and immune responses, while KEGG pathway analysis showed enrichment of gene clusters of associated with HPV infection and MAPK signaling. Of the differentially expressed genes, IFI6, SLC39A9 and ZNF185 showed a strong correlation with tumor progression and patient survival in the OncoLnc database while roles for AKAP12 and DUSP5 in carcinogenesis and poor prognosis have previously been established for other cancer types. Our study identified several novel HPV16 E7-regulated candidate genes with putative functions in tumorigenesis, thus providing new insights into HPV persistence in keratinocytes and early onset of tumorigenesis.

## Introduction

Cervical cancer ranks fourth globally among the most abundant female malignancies, with about 530,000 new cases and 270,000 deaths each year. It has been estimated that 85% of cervical cancer deaths occur in underdeveloped or developing countries, and mortality rates in low- and middle-income countries are 18 times higher than those in richer countries (Small et al., 2017). It is well-established that cervical cancer is associated with persistent human papillomavirus (HPV) infections and high-risk HPV types can be detected in 99.7% of the cervical cancer cases (Tummers and Burg, 2015).

Human papillomavirus are a family of small, non-enveloped viruses containing a circular double-stranded DNA genome. Six early genes (E1, E2, E4, E5, E6 and E7) play distinct roles in viral replication and cell transformation. E6 and E7 have shown to interact with various host proteins and carry out many modulatory functions (Moody and Laimins, 2010) as well as to block negative regulators of the cell cycle within the infected cell. The majority of HPV infections are asymptomatic and self-resolving. Once HPVs evade host immune defenses and establish persistence in basal keratinocytes, genetic alterations will accumulate and cells infected with high-risk HPV types can ultimately be driven into invasive cancer cells (Crosbie et al., 2013; Westrich et al., 2017) and early unapparent infections will finally develop into late-stage clinical neoplasm. However, it is still unclear which pre-cancerous lesions might progress (Schiffman et al., 2016).

Among high-risk HPVs, the most common type is HPV16 (Smith et al., 2007) and it has been shown that E7 plays an important role in the process of cellular proliferation and cell-cycle progression (Moody and Laimins, 2010). HPV16 E7 promotes the degradation of retinoblastoma protein (Rb), which leads to oncogenic transformation (Boyer et al., 1996). HPV displays a high affinity for mucosal and cutaneous epithelia and only infects basal proliferating keratinocytes (Doorbar, 2006). In addition, the HPV infectious life cycle is closely restricted by the differentiation state of the infected host cells (Doorbar et al., 2012), suggesting that primary human keratinocytes are the natural host for HPV infection. Therefore, primary normal human epidermal keratinocytes (NHEKs) are considered the ideal cell model for studying the mechanisms of HPV infection.

Digital RNA sequencing is a high-throughput sequencing method that is able to remove amplification biases. When constructing a sequencing library, PCR amplification steps are usually required to reach sufficient yields. However, target-sequence-dependent preferences during PCR amplification may lead to different amplification multiples of each target sequence. What is generated by PCR amplification is called duplication (Aird et al., 2011). The existence of duplication in traditional RNA sequencing will cause deviation between the quantitative results of subsequent gene expression and the true abundance of the sequence. However, during digital RNA sequencing molecular tags (UMI; unique molecular identifier) are added to unique mRNA transcripts, which prevent duplication and improves the accuracy of transcriptome quantification.

In this study, HPV16 E7-transfected NHEKs were investigated by digital RNA sequencing to identify novel genes, functions and signal transduction pathways to further explore E7-dependent mechanisms involved in HPV16 infection.

## Materials and Methods

### Cell Culture and Cell Transfection

HPV16-containing plasmid DNA (ATCC:45113D) was used as the template for PCR amplification and cloning of the HPV16 E7 gene into vector pEGFP-C1 following protocols described previously. NHEKs (ScienCell, Carlsbad, CA, United States) were cultured in 6-well plates using EpiLife culture medium (Gibco, United States) with 10% fetal bovine serum (Gibco, United States) and Human Keratinocyte Growth Supplement (Gibco, United States). NHEKs at 80% confluence were transfected with pEGFP-16E7 and pEGFP-C1 using Lipofectamine 3000 reagent (Invitrogen, Carlsbad, CA, United States). Six hours after Transfection, culture medium was replaced with fresh culture medium.

### RNA Isolation and Digital RNA Sequencing

Total RNA was isolated and purified using TRIzol reagent (Invitrogen, LifeTechnologies, United States) following the manufacturer’s procedures. RNA yields and sample purity were quantified using NanoDrop ND-1000 (NanoDrop, Wilmington, DE, United States). Digital RNA sequencing was conducted by LC-BIO Technologies Co., Ltd (Hangzhou, China). Each fragment was tagged with UMI tags (Islam et al., 2014). In the process of amplification, the products sharing the same UMI tag were merged and incorrect bases were corrected by multiple sequence alignment, thus effectively removing the duplication and retaining the true gene expression (Shiroguchi et al., 2012; Pflug and von Haeseler, 2018).

### Cluster Analysis of Differentially Expressed Genes

By grouping genes with the same or similar expression patterns, the function of unknown genes or unknown functions of known genes can be identified. In order to visualize the gene clustering expression pattern, the Fragments Per Kilobase of transcript per Million mapped reads (FPKM) value of the differential gene was transformed with the *Z* value (see the formula below) to draw a clustering diagram.

$$\begin{array}{c} Z\;\mathrm{sample}-i\;=\;[(\log2(\mathrm{Signal}\;\mathrm{sample}-i)-\mathrm{Mean}\;(\mathrm{Log}2\\ \;(\mathrm{Signal})\;\mathrm{of}\;\mathrm{all}\;\mathrm{samples})][\mathrm{Standard}\;\mathrm{deviation}\;(\mathrm{Log}2(\mathrm{Signal})\\ \;\mathrm{of}\;\mathrm{all}\;\mathrm{samples})] \end{array}$$

### Gene Ontology (GO) Enrichment Analysis

Gene Ontology annotation is divided into three primary categories: Biological process, Cellular component and Molecular function, and explains the biological role of genes from different perspectives. GO functional significance enrichment analysis was firstly mapped on all significant differentially expressed genes to each term of the Gene Ontology database, then the number of genes in each term was calculated and a hypergeometric test was applied to find significantly enriched GO items compared with the whole genomic background. The histogram and scatterplot of GO enrichment analysis results reflected the number distribution of differentially expressed genes on the GO term enriched in Biological processes, Cell components and Molecular functions.

### KEGG Pathway Enrichment Analysis

*In vivo*, different genes coordinate with each other to perform their biological functions. KEGG pathway enrichment analysis can determine the most important biochemical metabolic pathways and signal transduction pathways in which differentially expressed genes participate. The abundant pathway information in the KEGG database helps to understand the biological functions of genes from a systematic level, such as metabolic pathways, transmission of genetic information and some complex biological functions such as cell processes.

### Immunofluorescence Assays

HPV16 E7-transfected and control NHEKs were prepared for immunofluorescence assays at 80% confluence. Cells were washed with PBS and fixed with 4% paraformaldehyde (Solarbio, Beijing, China) for 20 min at room temperature. The fixed cells were washed and incubated in 0.5% (v/v) Triton X-100 (Solarbio, Beijing, China) for 10 min. Subsequently, cells were blocked with 10% goat serum in 1% bovine serum albumin (BSA) for an hour at room temperature and incubated overnight at 4°C with rabbit polyclonal anti-HPV16 E7 antibody (1:800 dilution), which was prepared by our laboratory previously (Zheng et al., 2016). Cells were subsequently washed and incubated with Alexa Fluor 594-conjugated secondary donkey anti-rabbit IgG (1:400 dilution, Yeasen, Shanghai, China) for an hour at 37°C in a dark box. Finally, the cell nuclei were stained with 4,6- diamidino-2-phenylindole (DAPI, 1:1000 dilution, Abcam) for 10 min at room temperature. Cells were observed under a fluorescence microscope (Olympus cellSens 2.3).

### Quantitative Real-Time PCR Analysis

cDNA was generated from the total RNA using the ReverTra Ace^®^ qPCR RT Master Mix (TOYOBO, JAPAN). TB Premix Ex TaqTM (Takara, Japan) was used for quantitative real-time PCR analysis according to the manufacturer’s protocols. Each reaction was run in triplicate. The relative gene expression levels were normalized with human beta-actin as internal reference following the 2-ΔΔCT method. Primer sequences are provided in Supplementary Table S1.

### Survival Rate Analysis

OncoLnc public database^1^ is a comprehensive interactive tool which contains patient survival data of 21 cancer types from TCGA projects. The patients were divided into 2 subgroups according to gene expression levels by setting the lower and higher percentile to 20. In this study, we analyzed the relationship between specific gene expressions and overall survival in cervical squamous cell carcinoma and endocervical adenocarcinoma (CESC). The patient information including Case ID, Days to last follow up, Status, Expression Group, race, age at diagnosis and primary diagnosis are shown in the Supplementary Table S2.

### Western Blot Analysis

Proteins were extracted from corresponding NHEKs using RIPA buffer (Fdbio science, Hangzhou, China) containing 1% PMSF for 15 min, followed by centrifugation for 10 min at 14,000 rpm at 4°C. The protein content was measured with a BCA Protein Assay kit (Fdbio science, Hangzhou, China). After denaturing at 100°C for 10 min, protein samples were separated on a SDS-polyacrylamide gel and transferred to a polyvinylidene fluoride (PVDF, Bio-Rad) membrane. After blocking with 5% skimmed milk in Tris-Buffered Saline containing 0.5% (v/v) Tween-20 (TBST), the membrane was incubated with primary antibody at 4°C overnight. The commercial antibodies used as primary antibody were anti-AKAP12 (ab49849, 1:1000; Abcam, Cambridge, MA, United States), anti-DUSP5 (ab200708,1:1000; Abcam, Cambridge, MA, United States) and anti-beta actin (abs137975,1:5000; Absin, Shanghai, China). After washing with TBST, the membrane was further incubated with HRP-labeled goat anti-mouse IgGs (A0216, 1:5000; Beyotime, Shanghai, China) or HRP-labeled goat anti-rabbit IgGs (A0208, 1:5000; Beyotime, Shanghai, China) at 37°C for 2 h. Beta actin was used as a loading control for Western blotting.

## Results

### Digital RNA Sequencing of HPV16 E7-Transfected NHEKs

**FIGURE 1:**
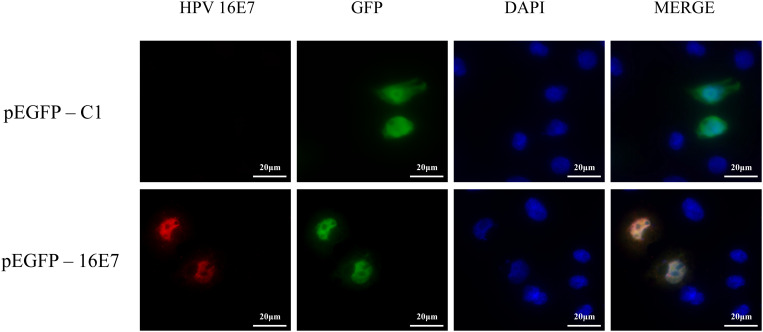
Verification of E7 expression in HPV16 E7-transfected NHEKs. NHEKs were transfected with the E7-expression vector pEGFP-16E7 or the control vector pEGFP-C1. E7 was detected by immunostaining **(red)** and the cell nucleus was stained with DAPI **(blue)**.

**FIGURE 2:**
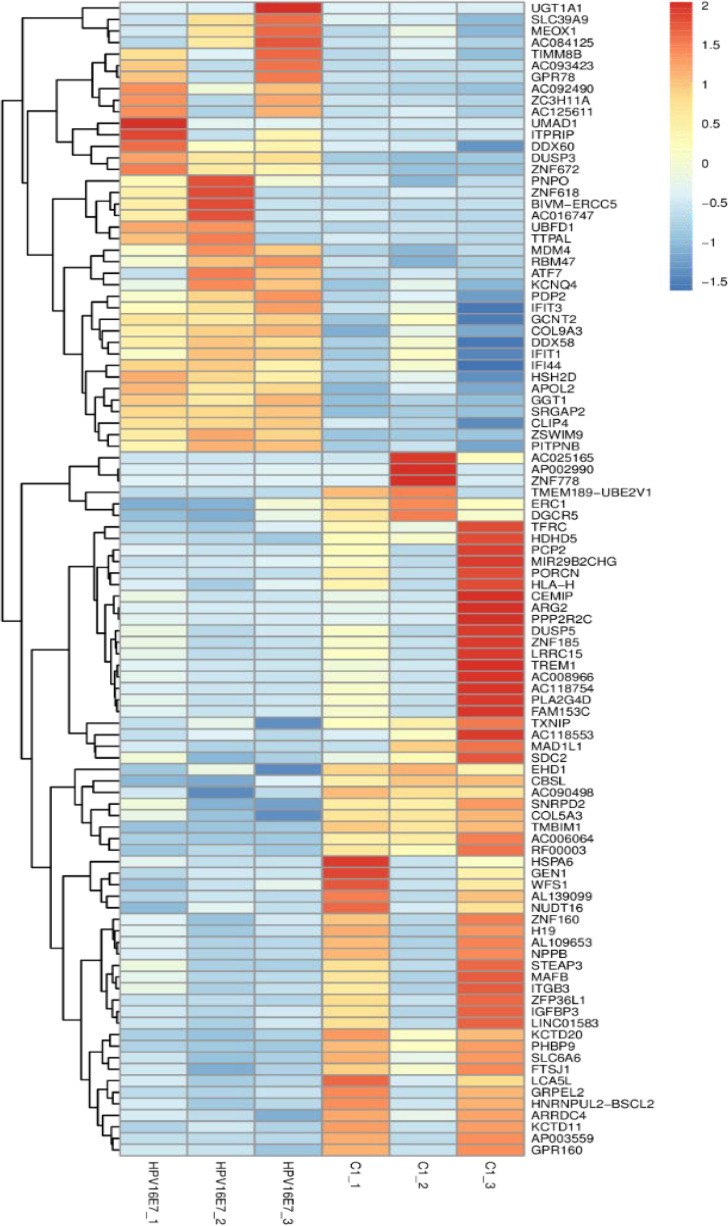
Heatmap of differentially expressed genes in HPV16 E7-transfected NHEKs and control cells. Colors represent differences in gene expression levels. HPV16E7_1, HPV16E7_2, and HPV16E7_3 represent the HPV16 E7-transfected samples and C1_1, C1_2, and C1_3 represent the control samples.

NHEKs were transfected with pEGFP-16E7 as the experimental group and with pEGFP-C1 as the control group, and E7 expression was confirmed by immunofluorescence (Figure 1). To identify novel E7-dependent genes and pathways involved in HPV16 infection, transfected cells were analyzed by digital RNA sequencing. Differentially expressed genes were selected based on the criteria “*p* < 0.05” and “[logFC] > 1,” which resulted in the identification of 195 significantly differentially expressed genes between the experimental and control group, including 62 up-regulated genes and 133 down-regulated genes in the experimental group. A number of differentially expressed genes between the HPV16 E7-expressing NHEKs and control cells were visualized in a heat map (Figure 2), which showed overall good consistency between replicates. The complete list of all 195 genes is shown in Supplementary Table S3.

### GO Enrichment Analysis

**FIGURE 3:**
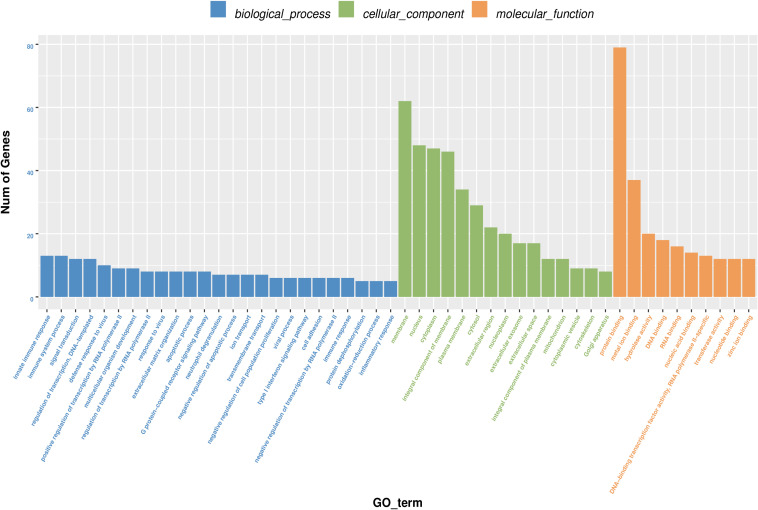
Gene Ontology (GO) terms classification of differentially expressed genes in HPV16 E7-transfected NHEKs. Blue columns represent biological process, green columns represent cellular component, and orange columns represent molecular function. Vertical axis displays the number of genes classified in each GO terms.

**FIGURE 4:**
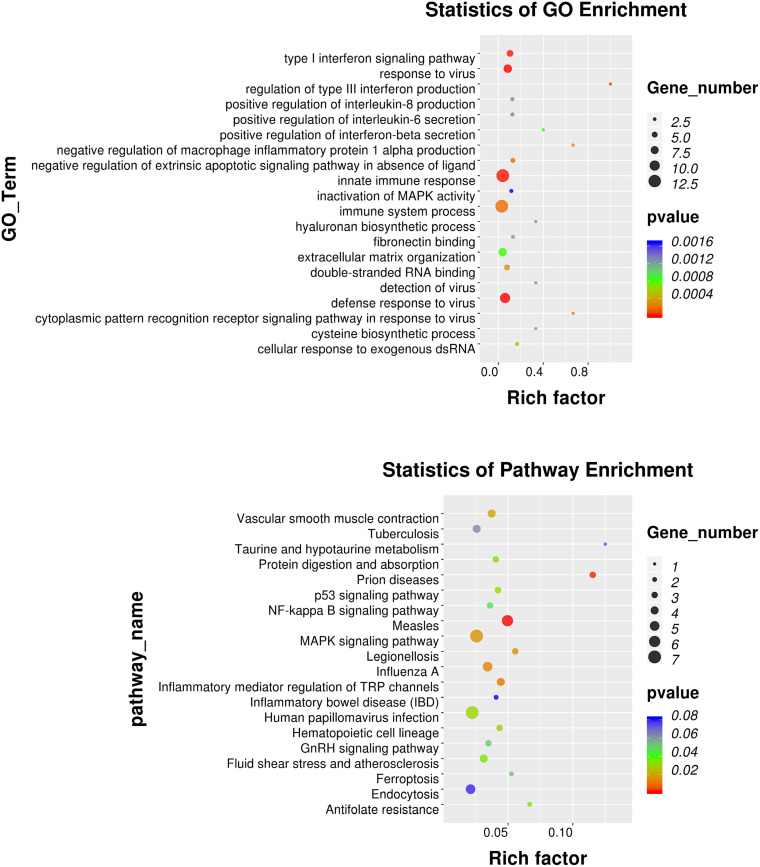
GO and KEGG pathway enrichment analysis. Scatter plot of the 20 most significant GO terms and KEGG pathways. The dot size indicates the number of differentially expressed genes enriched in the corresponding GO term/KEGG pathway. Different *p*-values are indicated by dot colors. The Rich factor is the ratio of the differentially expressed gene number to the total number of genes in the GO tern/KEGG pathway.

GO annotation analysis was used to analyze the 195 significantly differentially expressed genes to explore their potential biological function. GO terms were classified into three primary categories: Biological process, Cellular component and Molecular function (Figure 3). In biological process, most of the GO terms are related to virus infection and immune response. In the cellular component category, membrane, nucleus and cytoplasm are the top three components among others and we assume that HPV infection is a comprehensive process with gene pattern change happening in the whole cell. The greatest change in gene expression clustered in molecular function is protein binding, which may indicate the importance of protein-protein interactions during HPV infection. The 20 GO terms with the most significant *p*-value were displayed in a scatter plot representing the rich factor (ratio of the number of differentially expressed genes to the total number of genes in the GO term or pathway) and number of differentially expressed genes (Figure 4). It appears that HPV16 E7 induced strong anti-viral immune responses, since most differentially expressed genes were related to a variety of direct virus-related responses, such as response to virus (GO: 0009615), defense response to virus (GO:0051607), detection of virus (GO: 0009597), double-stranded RNA binding (GO: 0003725), and cellular response to exogenous dsRNA (GO: 0071360), and to immune responses, such as innate immune response (GO: 0045087), immune system process (GO: 0002376), and type I interferon signaling pathway (GO: 0060337). Furthermore, approximately one third of the differentially expressed genes seem to encode proteins with a membrane-related function and proteins displaying protein- or metal-binding activity.

### KEGG Pathway Enrichment Analysis

KEGG pathway enrichment analysis identifies pathways that are particularly abundant among the differentially expressed genes. The 20 KEGG pathways with the most significant p value were plotted in a scatter plot (Figure 4), which showed that the HPV infection (ko05165), with seven differentially expressed genes (ATP6V0A4, COL9A3, FZD8, HES2, ITGB3, OASL, and PPP2R2C), and MAPK signaling pathway (ko04010), also with seven differentially expressed genes (DUSP1, DUSP3, DUSP5, HSPA1A, HSPA6, IL1B, and PLA2G4D) were the most significant pathways. Furthermore, several pathways associated with other viral or intracellular bacterial infectious diseases showed numerous differentially expressed genes, such as Measles (ko05162), Influenza A (ko05164), Tuberculosis (ko05152) and Legionellosis (ko05134). However, the association of these pathways might in part be explained by overlapping genes, since IL1B, HSPA1A, and HSPA6 belong to almost all of these KEGG pathways. Nevertheless, it appears that transfection with only HPV16 E7 already induced a specific response against HPV and other intracellular viral or bacterial pathogens.

### Analyses of Differentially Expressed Genes

**FIGURE 5:**
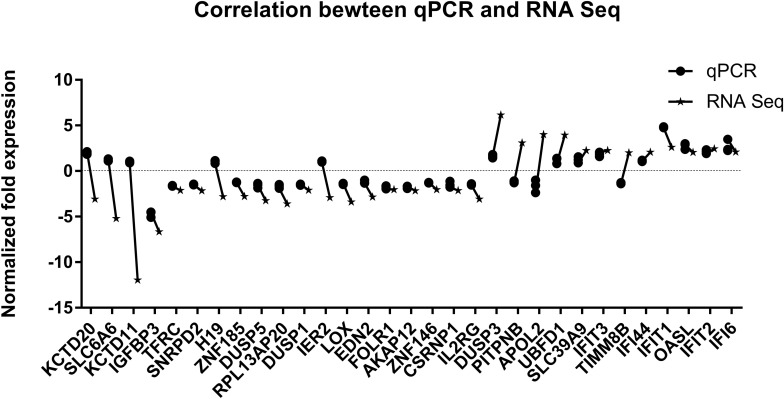
Validation of RNA sequencing data by quantitative real-time PCR. The 31 most significant differentially expressed genes displaying high expression abundance were selected.

Since differential expression in low-expressing genes is easily affected by confounding factors, genes with an FPKM value < 10 were excluded from further analyses. A total of 31 genes with relatively high expression abundance (the average gene expression abundance or at least one group of samples was >10) were selected for further analyses (Table 1). Among these 12 up-regulated and 19 down-regulated genes, 23 genes showed consistent differential expression results between qRT-PCR analyses and RNA-seq data (Figure 5). DUSP3, UBFD1, SLC39A9, IFIT3, IFI44, IFIT1, OASL, IFIT2 and IFI6 are upregulated while IGFBP3, TFRC, SNRPD2, ZNF185, DUSP5, RPL13AP20, DUSP1, LOX, EDN2, FLOR1, AKAP12, ZNF146, CSRNP1, and IL2RG are downregulated in mRNA level.

**TABLE 1:** Selected 31 differentially expressed genes with high expression abundance in HPV16 *E7*-transfected NHEKs.

Gene name	Description	Fold change	*P* value	Regulation Trend
AC006064	Novel transcript, antisense to GAPDH	0.02	<0.01	Down
KCTD20	Potassium channel tetramerization domain containing 20 [Source:HGNC Symbol; Acc:HGNC:21052]	0.32	<0.01	Down
SLC6A6	Solute carrier family 6 member 6 [Source:HGNC Symbol; Acc:HGNC:11052]	0.19	<0.01	Down
KCTD11	Potassium channel tetramerization domain containing 11 [Source:HGNC Symbol; Acc:HGNC:21302]	0.08	<0.01	Down
IGFBP3	Insulin like growth factor binding protein 3 [Source:HGNC Symbol; Acc:HGNC:5472]	0.15	<0.01	Down
TFRC	Transferrin receptor [Source:HGNC Symbol; Acc:HGNC:11763]	0.47	<0.01	Down
SNRPD2	Small nuclear ribonucleoprotein D2 polypeptide [Source:HGNC Symbol; Acc:HGNC:11159]	0.47	<0.01	Down
H19	H19, imprinted maternally expressed transcript [Source:HGNC Symbol; Acc:HGNC:4713]	0.36	<0.01	Down
ZNF185	Zinc finger protein 185 with LIM domain [Source:HGNC Symbol; Acc:HGNC:12976]	0.36	0.01	Down
AC090498	Ribosomal protein L41 (RPL41) pseudogene	0.48	0.01	Down
DUSP5	Dual specificity phosphatase 5 [Source:HGNC Symbol; Acc:HGNC:3071]	0.31	0.02	Down
RPL13AP20	Ribosomal protein L13a pseudogene 20 [Source:HGNC Symbol; Acc:HGNC:35709]	0.28	0.02	Down
DUSP1	Dual specificity phosphatase 1 [Source:HGNC Symbol; Acc:HGNC:3064]	0.48	0.02	Down
IER2	Immediate early response 2 [Source:HGNC Symbol; Acc:HGNC:28871]	0.34	0.02	Down
LOX	Lysyl oxidase [Source:HGNC Symbol; Acc:HGNC:6664]	0.30	0.02	Down
EDN2	Endothelin 2 [Source:HGNC Symbol; Acc:HGNC:3177]	0.35	0.03	Down
FOLR1	Folate receptor 1 [Source:HGNC Symbol; Acc:HGNC:3791]	0.49	0.03	Down
AP001453	Novel transcript	0.45	0.03	Down
AKAP12	A-kinase anchoring protein 12 [Source:HGNC Symbol; Acc:HGNC:370]	0.46	0.04	Down
HSPA1A	Heat shock protein family A (Hsp70) member 1A [Source:HGNC Symbol; Acc:HGNC:5232]	0.47	0.04	Down
ZNF146	Zinc finger protein 146 [Source:HGNC Symbol; Acc:HGNC:12931]	0.49	0.04	Down
ZNF664	Zinc finger protein 664 [Source:HGNC Symbol; Acc:HGNC:25406]	0.45	0.04	Down
CSRNP1	Cysteine and serine rich nuclear protein 1 [Source:HGNC Symbol; Acc:HGNC:14300]	0.47	0.04	Down
IL2RG	Interleukin 2 receptor subunit gamma [Source:HGNC Symbol; Acc:HGNC:6010]	0.33	0.05	Down
DUSP3	Dual specificity phosphatase 3 [Source:HGNC Symbol; Acc:HGNC:3069]	6.15	<0.01	Up
PITPNB	Phosphatidylinositol transfer protein beta [Source:HGNC Symbol; Acc:HGNC:9002]	3.10	<0.01	Up
APOL2	Apolipoprotein L2 [Source:HGNC Symbol; Acc:HGNC:619]	4.01	<0.01	Up
UBFD1	Ubiquitin family domain containing 1 [Source:HGNC Symbol; Acc:HGNC:30565]	3.94	<0.01	Up
SLC39A9	Solute carrier family 39 member 9 [Source:HGNC Symbol; Acc:HGNC:20182]	2.26	0.01	Up
IFIT3	Interferon induced protein with tetratricopeptide repeats 3 [Source:HGNC Symbol; Acc:HGNC:5411]	2.26	0.01	Up
TIMM8B	Translocase of inner mitochondrial membrane 8 homolog B [Source:HGNC Symbol; Acc:HGNC:11818]	2.02	0.01	Up
IFI44	Interferon induced protein 44 [Source:HGNC Symbol; Acc:HGNC:16938]	2.07	0.01	Up
IFIT1	Interferon induced protein with tetratricopeptide repeats 1 [Source:HGNC Symbol; Acc:HGNC:5407]	2.61	0.01	Up
OASL	2’-5’-oligoadenylate synthetase like [Source:HGNC Symbol; Acc:HGNC:8090]	2.06	0.02	Up
IFIT2	Interferon induced protein with tetratricopeptide repeats 2 [Source:HGNC Symbol; Acc:HGNC:5409]	2.44	0.02	Up
IFI6	Interferon alpha inducible protein 6 [Source:HGNC Symbol; Acc:HGNC:4054]	2.10	0.05	Up

### Correlation of Differentially Expressed Genes With Cancer Survival

**FIGURE 6:**
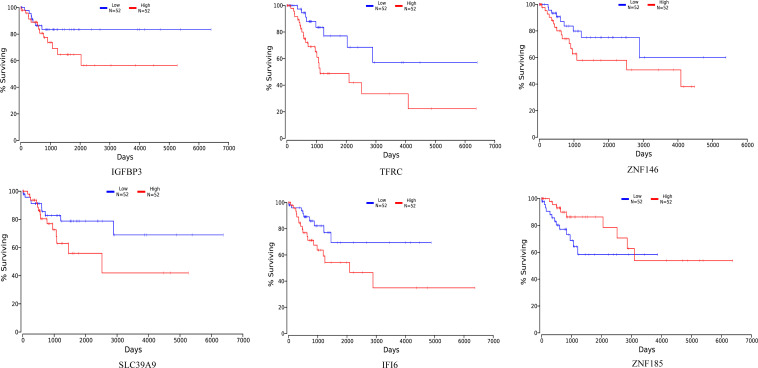
Correlation of between differentially expressed genes in HPV16 E7-transfected NHEKs and cancer patient survival using the OncoLnc database. The red curves display patient survival when expression of IGFBP3, TFRC, ZNF185, ZNF146, SLC39A9 or IFI6 is high, while the blue curves display survival when expression is low.

The relation between the 23 differential expressed genes and cancer survival was analyzed using OncoLnc public database (Anaya, 2016). Six genes were identified from the surviving analysis to have a significant impact on cancer survival (Figure 6). HPV16 E7-transfected cells showed reduced expression of IGFBP3, TFRC and ZNF146, however, lower expression of these genes was correlated with increased survival. In contrast, SLC39A9 and IFI6 were induced in HPV16 E7-transfected cells, while for these genes high expression was correlated with reduced survival. Finally, ZNF185 expression was reduced in HPV16 E7-transfected cells, while low expression is correlated with reduced survival. Thus far, these genes have not yet been studied in relation with HPV infections.

### AKAP12 and DUSP5 Protein Expression

**FIGURE 7:**
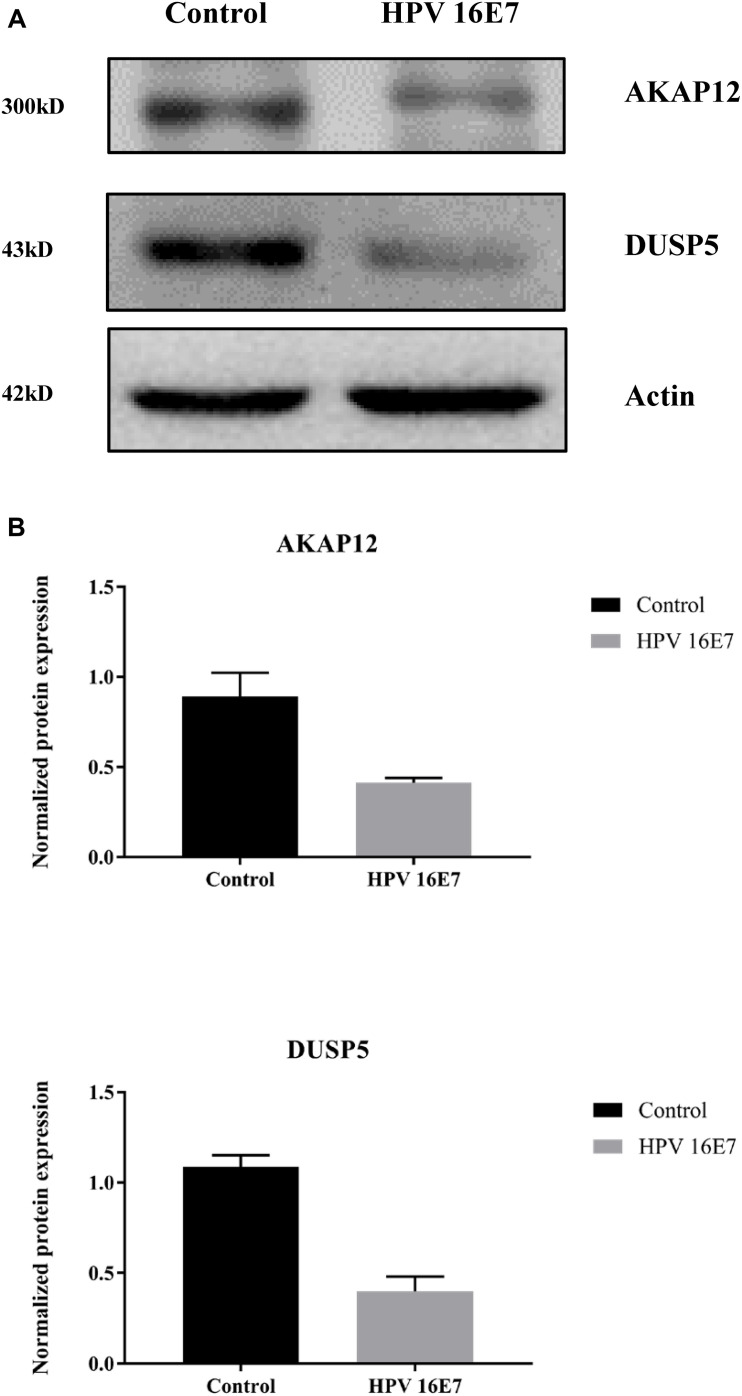
Validation of AKAP12 and DUSP5 protein expression levels in HPV16 E7-transfected NHEKs. **(A)** Western analysis of AKAP12 and DUSP5 in HPV16 E7–transfected NHEKs and control cells. **(B)** Quantification of AKAP12 and DUSP5 protein levels. The levels were normalized to β-Actin levels.

In our previous study (Zhang et al., 2018), we used keratinocytes to overexpress E7 from HPV type 6b and 16 and identified that AKAP12 was downregulated, which is consistent with AKAP12 expression in the current study. Also, KEGG pathway enrichment analysis showed that the MAPK signaling pathway was the most clustered signaling pathway and among the seven genes enriched in MAPK signaling pathway low DUSP5 expression has already been clearly established as a correlate for carcinogenesis and poor prognosis. Therefore, protein expression levels of AKAP12 and DUSP5 were further verified by Western analysis. Indeed, protein levels were significantly reduced in HPV16 E7-transfected cells compared with the control cells (Figure 7).

## Discussion

In this study, NHEKs were transfected with a high-risk HPV type 16 E7-expression vector or control vector and expression profiles were analyzed by digital RNA sequencing. Inevitable random errors in the sequencing process and automated library construction which may be amplified to a certain extent, as well as the relatively low transfection efficiency of primary keratinocytes cause the variability between the intra-groups. In addition, our study focuses on early changes of host cell with HPV 16E7, since relatively low transfection efficiency may reflect natural infection, further selection is not included considering it may maximize or minimize some effects of related genes. As a result, we identified 23 potential candidates among 195 differentially expressed genes. Nevertheless, it is well acknowledged that the inhibition of Rb family which controls the activity of E2F transcription factors for G1-S cell cycle by E7 is important for cellular proliferation and progression to precancer (Moody and Laimins, 2010), the missing of some cell cycle genes such as p16 in the list may mainly due to our transient transfection method which leads to a higher proportion of normal keratinocytes comparing with HPV 16E7 positive cells thus minimizing the effects of these genes. What’s more, since no human cancer arises as the acute consequence of infection, the latency periods between infected cells and invasive cancer cells are frequently in the range of 15–40 years (Zur, 2009), it is necessary to further study the role of E7 in HPV-induced persistence and the antiviral defense of host cell in escaping the malignant transformation.

Integrated analysis of the differentially expressed genes showed that nine of the 23 consistent differentially expressed genes were related to immunity. Further studies of virus-mediated immune dysregulation would also be critical for preventive and therapeutic tools of elimination of virus-infected cells. IFIT1, IFIT2, IFIT3 and IFI44 are all interferon-induced proteins that were up-regulated in HPV16 E7-transfected cells, which is consistent with previous studies on HPV infection (Boccardo et al., 2010; Kaczkowski et al., 2012; Ambuhl et al., 2017; Klymenko et al., 2017). These genes are commonly regarded as viral restriction factors and those immune alterations are critical for the prevention of HPV-infected cells during cancer progression. The up-regulated gene OASL is also involved in interferon gamma and cytokine signaling. A recent study showed that OASL expression is relatively high in HPV positive cervical cancer, and seemed to be related with resistance to cisplatin (Zhang et al., 2019a). According to the NCBI RefSeq database, the product of the down-regulated gene IGFBP3 forms a ternary complex with insulin-like growth factor acid-labile subunit (IGFALS) and either insulin-like growth factor (IGF) I or II. In this form, it circulates in the plasma, prolonging the half-life of IGFs and altering their interaction with cell surface receptors. It has previously been shown that high-level expression of IGFBP3 stimulated growth inhibition of tumors in an HPV16 E7-induced cervical cancer model (Musil et al., 2014). Furthermore, HPV16 early gene depletion induced expression of IGFBP3 (Hanning et al., 2013). Therefore, HPV16 E7-induced repression of IGFBP3 might be related with onset of carcinogenesis. The down-regulated gene TFRC encodes a transferrin receptor and plays an important role in the uptake of cellular iron. But HPV-mediated immune alteration analysis using The Cancer Genome Atlas shows that upregulation of TFRC may relate to a poor prognosis of survival of cervical cancer patients (Chen et al., 2018). The down-regulated gene IL2RG encodes an important signaling component of many interleukin receptors, and IL2RG deficiency can result in differential signaling defects (Illig et al., 2019) while its mutation may cause immunodeficiency (Hsu et al., 2015). The high abundance of immunity-related differentially expressed genes indicates that HPV16 E7-mediated immune dysregulation might be important to prevent the elimination of HPV from infected cells during virus persistence.

Three of the differentially expressed genes belong to the dual-specificity protein phosphatase subfamily, with DUSP1 and DUSP5 downregulated and DUSP3 upregulated. These negative regulators of the mitogen-activated protein (MAP) kinases show different substrate specificities for various MAP kinases and different modes of induction by extracellular stimuli. These genes appear to play an important role in cellular proliferation and differentiation as well as cellular response to environmental stress (Jeffrey et al., 2007; Patterson et al., 2009). For example, DUSP1-expressing Hela cells display a relatively low proliferation rate *in vitro* (Cheng et al., 2015), while DUSP3 interacts with high-expressing nucleolar proteins involved in DNA repair and senescence in Hela cells (Panico and Forti, 2013). As our data shows, the protein level of DUSP5 is reduced with HPV 16E7 overexpressing. And several studies showed that suppression of DUSP5 expression correlates with cancer cell proliferation, migration, invasion and poor prognosis in different cancer types (Liu et al., 2018; Wang et al., 2019; Du et al., 2020), but the relationship between DUSP5 and HPV has thus far remained undiscovered. Therefore, these genes may be the candidates for further studies.

The two downregulated genes ZNF185 and ZNF146 belong to the zinc finger protein family, which plays important roles in various cellular functions such as cell proliferation, differentiation, and apoptosis. A previous study showed that ZNF185 silencing strongly impaired keratinocyte differentiation in the head and neck, and in cervical and squamous cell carcinomas (Smirnov et al., 2019), however, functions for ZNF146 have remained unclear to date.

Other down-regulated genes, like LOX, EDN2, FOLR1, and CSRNP1, could not be categorized, although some distinct functions have previously been described. LOX encodes a member of the lysyl oxidase family proteins, which has previously been proposed to promote epithelial to mesenchymal transition in prostate cancer (Gonzalez-Chavarria et al., 2018), and its propeptide domain may display tumor suppressor activities (Agra et al., 2013). EDN2 belongs to the endothelin protein family of secretory vasoconstrictive peptides, and it has already been shown that it is involved in ovulation (Cacioppo et al., 2017). FOLR1 is a member of the folate receptor family, and a previous study showed it is associated with the progression of cervical cancer through the ERK signaling pathway (Liu et al., 2017). CSRNP1 is a key protein of the non-canonical Wnt pathway (Miyasaka et al., 2007), and because of its decreased level of expression in tumor tissues it was suggested to serve as a tumor suppressor (Ishiguro et al., 2001; Wang et al., 2013). Finally, two little-studied genes were identified, the upregulated pseudogene RPL13AP20 and the down-regulated gene SNRPD2, which may participate in lncRNA splicing (Azam et al., 2019).

Previous study has shown that AKAP12 expression is enhanced after *trans*-uterine arterial chemoembolization (TUACE) in cervical cancer patients (Chen et al., 2019). And we verified that AKAP12 is downregulated in both mRNA and protein level with HPV 16E7 overexpressing, so we deduce AKAP12 may play a role in HPV infection and further carcinogenesis.

HPV16 E7-mediated dysregulation of expression of target genes might be also the complex control of microRNAs (miRNA), long non-coding RNAs (lncRNA) and circular RNAs (circRNA) which are important to prevent the elimination of HPV from infected cells during virus persistence and the consequent tumorigenesis induction. For example, the lncRNA MALAT1 is overexpressed with high risk HPVs infection (Jiang et al., 2014; Yang et al., 2015) while the lncRNA MALAT1/miR-145-5p/AKAP12 axis has been verified in prostate cancer (Xue et al., 2018). In addition, both the luciferase reporter assay and western blot analysis showed DUSP5 could be repressed by miR-181a (Li et al., 2007). To date, several studies have verified LUCAT1/miR-181a axis (Zhang et al., 2019b), miR-181a-5p/TGFβI axis (Zhu et al., 2019) and miR-181a-5p/MMP14 axis (Shen et al., 2019) in cervical cancer. These might give new insights for further study.

## Conclusion

Most high-risk HPV infections are transient and will not progress to clinically significant cervical cancer with host immune responses generally able to clear the virus. In the process of persistent infections, gene alterations and changes in cellular phenotype will accumulate and finally drive the infected cells into invasive cancer cells. Cervical cancers are almost exclusively caused by high-risk HPV infections, and HPV16 accounts for the largest portion. HPV E7 has been reported to be closely related with cellular proliferation and oncogenic transformation. Therefore, HPV16 E7-transfected keratinocytes were studied to identify associated genes and pathways. A total of 23 consistent differentially expressed genes were identified, with several of them displaying high credibility for the association between E7 and carcinogenesis. Interestingly, the existence of alternative splicing isoforms of the E7 gene may influence E7 translation (Brant et al., 2019), which may explain some of the observed bias in transcript levels and diversity between biological repeats (Supplementary Figures S1a,b). Digital RNA sequencing allowed in-depth analysis of differential gene expression with high credibility. HPV16 E7 showed a large impact on immune-related networks in keratinocytes. AKAP12, DUSP5, IFI6 and SLC39A9 are five important novel candidate genes related to E7 expression for which future exploration into the molecular mechanisms. Overall, this study has laid the foundation for further studies exploring HPV16 E7 related mechanisms for clearance and persistence of HPV16 in human epidermal keratinocytes.

## Data Availability Statement

The raw datasets for this study can be found in the Gene Expression 317 Omnibus (https://www.ncbi.nlm.nih.gov/geo/query/acc.cgi?&acc=gse145224).

## Author Contributions

HC conceived the work and undertook the leadership. CH wrote the draft of the manuscript. CH, JZ, BZ, SS, and YS contributed to the acquisition and interpretation of the data. SV and HC revised and polished the manuscript. All the authors contributed to the manuscript revision, read and approved the submitted version.

## Conflict of Interest

The authors declare that the research was conducted in the absence of any commercial or financial relationships that could be construed as a potential conflict of interest.

## Footnotes

1 http://www.oncolnc.org

## Abbreviations

HPV: human papillomavirus
NHEKs: normal human epidermal keratinocytes.
