# Supplementary figures and images for: Digital RNA Sequencing of Human Epidermal Keratinocytes Carrying Human Papillomavirus Type 16 E7
Source: Front Genet. 2020 Aug 5;11:819. doi: 10.3389/fgene.2020.00819 (PMC7419603; doi:10.3389/fgene.2020.00819)

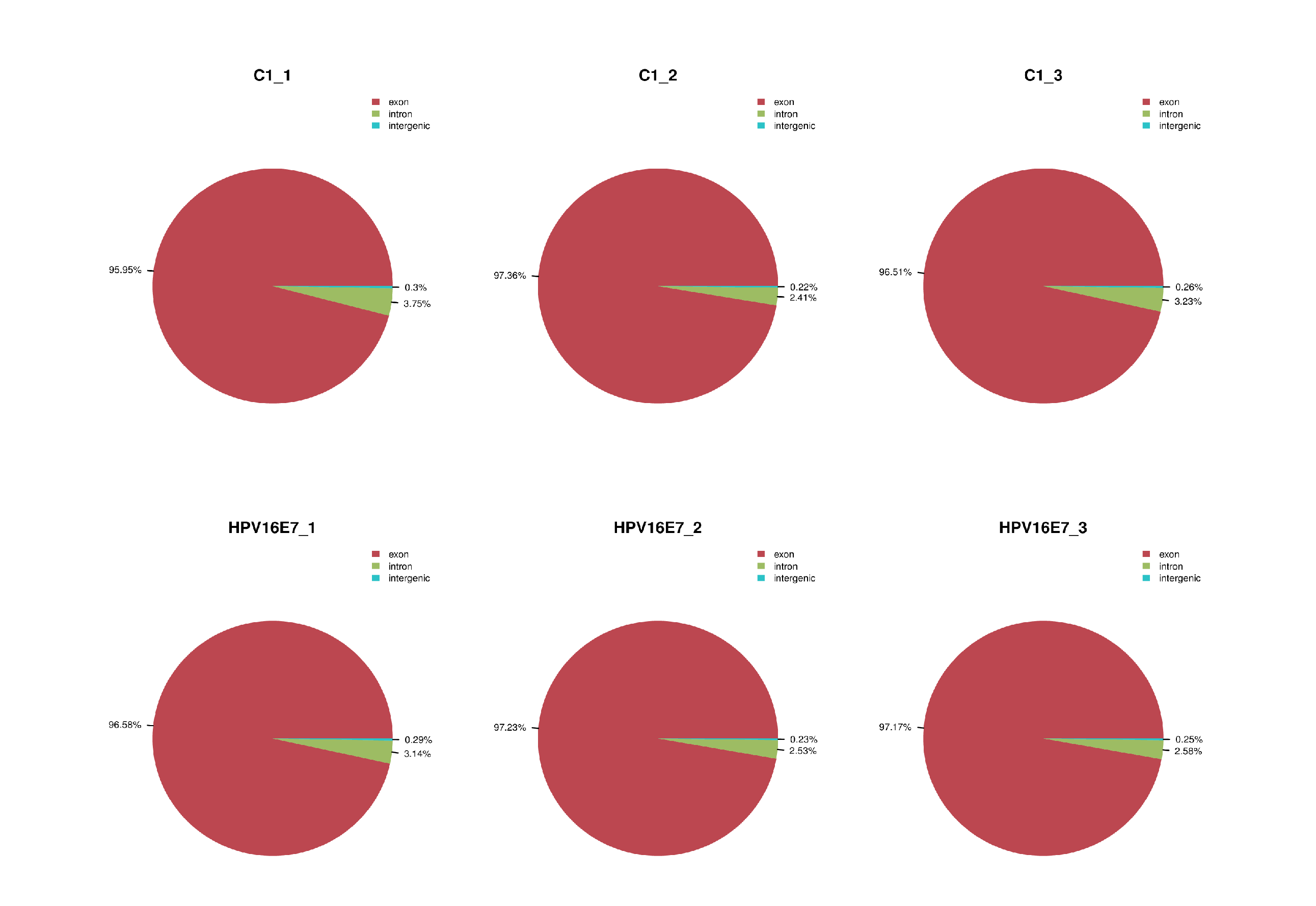

Supplement: Supplementary file 1 [file Image_1.TIF]

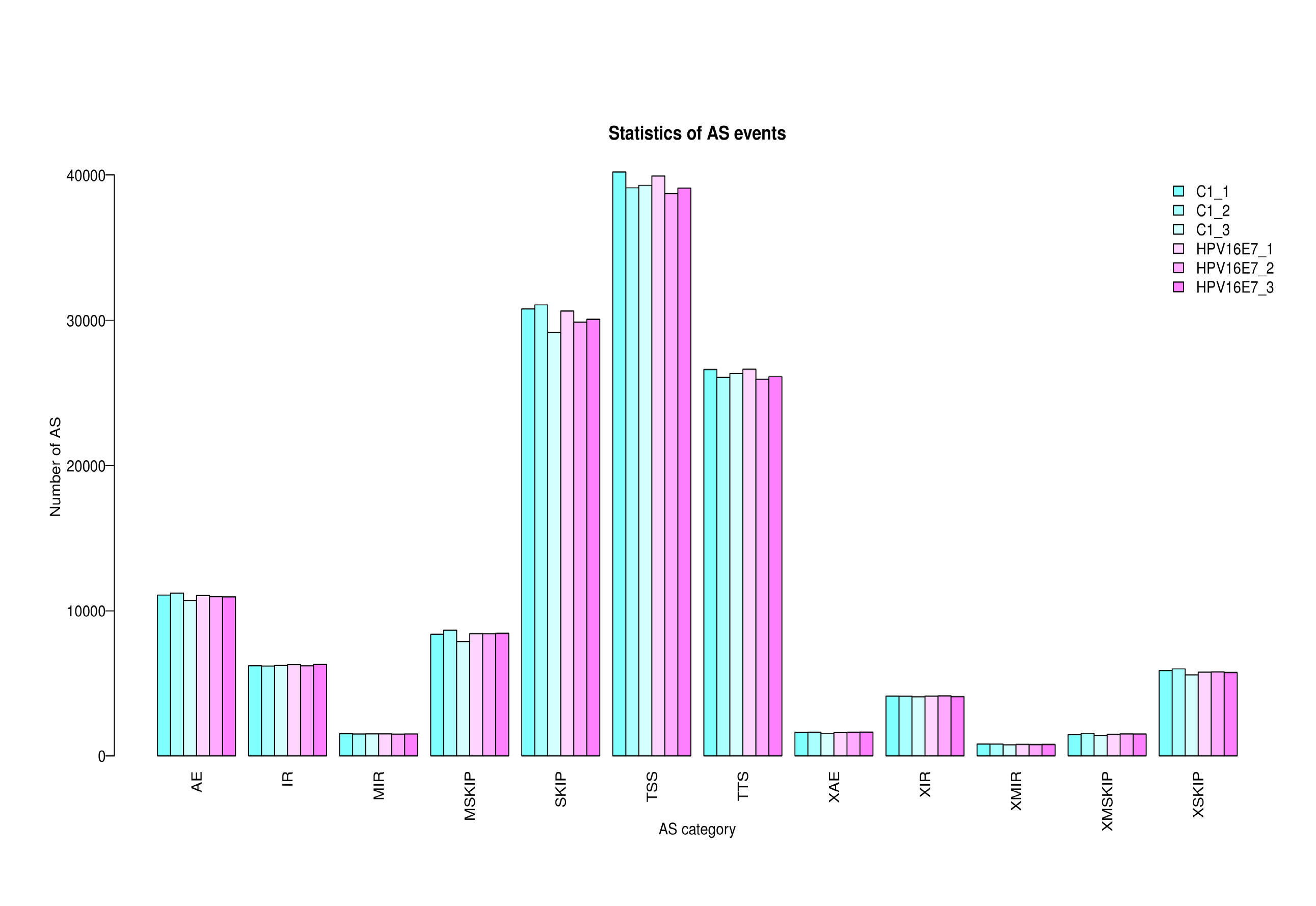

Supplement: Supplementary file 2 [file Image_2.TIF]
