# Supplementary material for: Digital RNA Sequencing of Human Epidermal Keratinocytes Carrying Human Papillomavirus Type 16 E7
Source: Front Genet. 2020 Aug 5;11:819. doi: 10.3389/fgene.2020.00819 (PMC7419603; doi:10.3389/fgene.2020.00819)
Supplement: Supplementary file 3 [file Table_1.DOC]

Supplementary Table S1**|** Primers pairs used in quantitative real-time PCR.

| **Gene symbol** | **Gene ID** | **Forward primers (5′-3′)** | **Reverse primers (5′-3′)** | **Size** |
| --- | --- | --- | --- | --- |
| KCTD20 | 222658 | TGACAGTGACAGGTTATTGCG | AGGCATAGTCAAGTGAGAGGTC | 161 |
| SLC6A6 | 6533 | GGAGAAGTGGTCTAGCAAGATCG | AGAAACGCACCTCCACCATTC | 117 |
| KCTD11 | 147040 | CGGGGACCCCATCACTATGA | TCAGAGTCGGTGCAGAAAAGG | 77 |
| IGFBP3 | 3486 | AGAGCACAGATACCCAGAACT | GGTGATTCAGTGTGTCTTCCATT | 93 |
| TFRC | 7037 | ACCATTGTCATATACCCGGTTCA | CAATAGCCCAAGTAGCCAATCAT | 219 |
| SNRPD2 | 6633 | AGTCAAGAACAATACCCAAGTGC | ATGTTGCAGTGCCTATCGAAG | 90 |
| H19 | 283120 | AGCAGCCTTCAAGCATTC | TGTTTATTGATGATGAGTCCAG | 94 |
| ZNF185 | 7739 | TTGGCTGATTATGAGGGGAAGG | TCTCTCGTCTGACAAGTTGCT | 181 |
| DUSP5 | 1847 | TGTCGTCCTCACCTCGCTA | GGGCTCTCTCACTCTCAATCTTC | 152 |
| RPL13AP20 | 387841 | GGTCCTGGTGCTTGATGG | CTGGCTTGGTCTTGTGGG | 269 |
| DUSP1 | 1843 | AGTACCCCACTCTACGATCAGG | GAAGCGTGATACGCACTGC | 77 |
| IER2 | 9592 | ACTGGTCCCGAGCAAGAAAG | CGACTTCGGATGACGCTCC | 68 |
| LOX | 4015 | CGGCGGAGGAAAACTGTCT | TCGGCTGGGTAAGAAATCTGA | 128 |
| EDN2 | 1907 | CGTCCTCATCTCATGCCCAAG | AGGCCGTAAGGAGCTGTCT | 142 |
| FOLR1 | 2348 | GCTCAGCGGATGACAACACA | CCTGGCCCATGCAATCCTT | 87 |
| AKAP12 | 9590 | GAGATGGCTACTAAGTCAGCGG | CAGTGGGTTGTGTTAGCTCTTC | 124 |
| ZNF146 | 7705 | ACCCCTTTGCCTGTAAGGTAT | TCTCTCTCGTGTGAAAATGCTC | 84 |
| CSRNP1 | 64651 | TGGCCGGTTGGAAGAAGTG | GCAGATCCTATCGCAGTGACAG | 166 |
| IL2RG | 3561 | GTGCAGCCACTATCTATTCTCTG | GTGAAGTGTTAGGTTCTCTGGAG | 187 |
| DUSP3 | 1845 | GGCTTTGGCTCAAAAGAATGG | AGGTAGGCGATAACTAGCGTT | 84 |
| PITPNB | 23760 | CTGTTCAGGAGTATCAGGTTGGG | GGCACTTTGCTCTTTAGGTGA | 166 |
| APOL2 | 23780 | CCGCAACTTGGACCAAAGC | CACGGATGTTCCTCCCAATC | 134 |
| UBFD1 | 56061 | TCTGGAATAAGACCAAGCATGAC | ACCTGTAATCGAGTGGATCTTCT | 86 |
| SLC39A9 | 55334 | CATGCTGGCTTAGAGCGGAAT | TGGTGCTGCCAATGCAAAGA | 63 |
| IFIT3 | 3437 | TCAGAAGTCTAGTCACTTGGGG | ACACCTTCGCCCTTTCATTTC | 201 |
| TIMM8B | 26521 | TCACTTCATGGAGTTATGTTGGG | AGACAATTTTCAGTGCGAGAGTC | 78 |
| IFI44 | 10561 | ATGGCAGTGACAACTCGTTTG | TCCTGGTAACTCTCTTCTGCATA | 212 |
| IFIT1 | 3434 | TTGATGACGATGAAATGCCTGA | CAGGTCACCAGACTCCTCAC | 214 |
| OASL | 8638 | CTGATGCAGGAACTGTATAGCAC | CACAGCGTCTAGCACCTCTT | 105 |
| IFIT2 | 3433 | CACATGGGCCGACTCTCAG | CCACACTTTAACCGTGTCCAC | 134 |
| IFI6 | 2537 | GGTCTGCGATCCTGAATGGG | TCACTATCGAGATACTTGTGGGT | 145 |
